# β-Adrenergic cAMP Signals Are Predominantly Regulated by Phosphodiesterase Type 4 in Cultured Adult Rat Aortic Smooth Muscle Cells

**DOI:** 10.1371/journal.pone.0047826

**Published:** 2012-10-18

**Authors:** Kui Zhai, Fabien Hubert, Valérie Nicolas, Guangju Ji, Rodolphe Fischmeister, Véronique Leblais

**Affiliations:** 1 Inserm UMR-S 769, LabEx LERMIT, Châtenay-Malabry, France; 2 Université Paris-Sud, Faculté de Pharmacie, Châtenay-Malabry, France; 3 National Laboratory of Biomacromolecules, Institute of Biophysics, Chinese Academy of Sciences, Beijing, China; 4 IPSIT IFR141, Plate-forme Imagerie Cellulaire, Châtenay-Malabry, France; University of Bonn, Germany

## Abstract

**Background:**

We investigated the role of cyclic nucleotide phosphodiesterases (PDEs) in the spatiotemporal control of intracellular cAMP concentrations in rat aortic smooth muscle cells (RASMCs).

**Methodology/Principal Findings:**

The rank order of PDE families contributing to global cAMP-PDE activity was PDE4> PDE3  =  PDE1. PDE7 mRNA expression but not activity was confirmed. The Fluorescence Resonance Energy Transfer (FRET)-based cAMP sensor, Epac1-camps, was used to monitor the time course of cytosolic cAMP changes. A pulse application of the β-adrenoceptor (β-AR) agonist isoproterenol (Iso) induced a transient FRET signal. Both β_1_- and β_2_-AR antagonists decreased the signal amplitude without affecting its kinetics. The non-selective PDE inhibitor (IBMX) dramatically increased the amplitude and delayed the recovery phase of Iso response, in agreement with a role of PDEs in degrading cAMP produced by Iso. Whereas PDE1, PDE3 and PDE7 blockades [with MIMX, cilostamide (Cil) and BRL 50481 (BRL), respectively] had no or minor effect on Iso response, PDE4 inhibition [with Ro-20-1724 (Ro)] strongly increased its amplitude and delayed its recovery. When Ro was applied concomitantly with MIMX or Cil (but not with BRL), the Iso response was drastically further prolonged. PDE4 inhibition similarly prolonged both β_1_- and β_2_-AR-mediated responses. When a membrane-targeted FRET sensor was used, PDE3 and PDE4 acted in a synergistic manner to hydrolyze the submembrane cAMP produced either at baseline or after β-AR stimulation.

**Conclusion/Significance:**

Our study underlines the importance of cAMP-PDEs in the dynamic control of intracellular cAMP signals in RASMCs, and demonstrates the prominent role of PDE4 in limiting β-AR responses. PDE4 inhibition unmasks an effect of PDE1 and PDE3 on cytosolic cAMP hydrolyzis, and acts synergistically with PDE3 inhibition at the submembrane compartment. This suggests that mixed PDE4/PDE1 or PDE4/PDE3 inhibitors would be attractive to potentiate cAMP-related functions in vascular cells.

## Introduction

In the vascular system, cAMP is a key physiological second messenger, which inhibits contraction, proliferation and migration of the smooth muscle cells (SMCs) [1], [2]. Intracellular concentration of cAMP is determined by the balance of its production by adenylyl cyclase and its degradation by specific enzymes, the 3′,5′-cyclic nucleotide phosphodiesterases (PDEs). PDEs are classified in 11 families based on structural similarity and enzymatic properties, including substrate specificity (cAMP *versus* cGMP), kinetic properties and regulation [3]. Within these PDE families, multiple isoforms are expressed, either as products of different genes or multiple transcriptional products of one gene. It is usually admitted that vascular SMCs express three dominant cAMP-PDE families (PDE1, PDE3 and PDE4), with a pattern of activity depending on the species, the vascular bed and the phenotype of the cell [4]. However, the expression/activity of more recently identified cAMP-PDEs (PDE7 to PDE11) has been poorly investigated. By comparing the mRNA expression of PDE1 to PDE10 in rat pulmonary and systemic vascular SMCs, Phillips *et al.* showed that PDE7 mRNA was expressed in all studied cells but PDE10 mRNA was never detected, whereas PDE8 and PDE9 mRNAs were differentially expressed depending on the vascular bed [5]. PDE11 was not examined in this study.

Such a multiplicity of PDE isoforms might seem functionally redundant. However, it is now well-accepted that cAMP is not uniformly distributed within cells so that its action may be restricted to subcellular domains of the cells, and that different signaling pathway components, including PDEs and cAMP-dependent protein kinase (PKA), may contribute to this phenomenon. This concept has been extensively developed in cardiac myocytes: the different cardiac PDE isoforms are targeted to distinct subcellular microdomains and contribute to the intracellular compartmentation of cAMP by limiting its diffusion to the entire cell, generating specific cardiac responses at discrete intracellular loci [6]–[8]. A similar picture of cardiac cyclic nucleotide compartmentation is also proposed for cGMP [6], [9]. By contrast, this concept has been poorly investigated in SMCs. In the case of cAMP signaling, Delpy *et* *al.* showed that, in rat endothelium-denuded aorta, PDE3 inhibition potentiates both the increase in intracellular cAMP level and the cAMP-dependent vasorelaxation elicited by β-adrenergic stimulation, whereas PDE4 inhibition only potentiates the former response without modifying the latter one [10]. The lack of correlation between the cAMP concentration and the functional response during PDE inhibition suggests the presence of distinct intracellular cAMP pools in vascular SMCs controlled by different PDE isoforms. However, these data obtained in an integrated tissue were never confirmed at the cellular level. In the case of cGMP signaling, recent studies provided evidence that in isolated vascular SMCs, nitric oxide and natriuretic peptides induce distinct cGMP signals, partly due to a differential regulation by PDE5 [11], [12].

Thus, the main objective of this study was to investigate the role of the different PDE families in the intracellular cAMP compartmentation of vascular SMCs. For this purpose, we took advantage of the Fluorescence Resonance Energy Transfer (FRET)-based imaging technique, using Epac-based sensors which allow a spatiotemporal monitoring of cAMP concentrations in intact living cells [13], [14]. As cellular model of vascular SMCs, we used rat aorta SMCs (RASMCs) maintained in culture. During cell culture, vascular SMCs undergo a phenotypic switch from a contractile/quiescent to a proliferative/synthetic phenotype, miming the phenotype of a cell isolated from an injured vessel [15]. It is known that this phenotypic switch is associated with modifications of PDE levels and activity, which depend on the species and may vary between culture conditions [16]–[18]. Thus, the first aim of this study was to characterize the expression and activity pattern of cAMP-PDE isoforms in cultured RASMCs. For this purpose, we performed reverse transcription-polymerase chain reaction (RT-PCR) experiments and a biochemical PDE activity assay coupled to a pharmacological approach by using selective PDE inhibitors. The second aim of this study was to delineate the role of PDEs in modulating the spatiotemporal dynamic of cAMP signals elicited in living RASMCs, by using FRET-based sensors targeted to either the cytosolic or the plasma membrane compartments. Isoproterenol (Iso), a β-adrenoceptor (β-AR) agonist, was used here as a physiological cAMP-elevating stimulus.

## Materials and Methods

All experiments performed conform to the European Community guiding principles in the care and use of animals (86/609/CEE, CE Off J no. L358, December 18, 1986), the local ethics committee (CREEA Ile-de-France Sud) guidelines, and the French decree no. 97–848 of October 19, 1987 (J Off République Française, October 20, 1987, pp 12245–12248). Authorizations to perform animal experiments according to this decree were obtained from the French Ministry of Agriculture, Fisheries and Food (no. 92–283, June 27, 2007).

### Pharmacological agents

CGP-20712A methanesulfonate salt (CGP), ICI 118,551 hydroclhoride (ICI), 3-isobutyl-1-methylxanthine (IBMX) and (-)-isoproterenol hydrochloride (Iso) were purchased from Sigma Aldrich (St Quentin, Fallavier, France). Cilostamide (Cil) and BRL 50481 (BRL) were from Tocris Bioscience (Bristol, UK), 8-methoxymethyl-3-isobutyl-1-methylxanthine (MIMX) and Ro-20-1724 (Ro) from Calbiochem (Merck Chemicals Ltd, Nottingham, UK), and BAY-60-7550 (BAY) from Cayman Chemical (Bertin Pharma, Montigny-le-Bretonneux, France). As all PDE inhibitors stock solutions were prepared in dimethylsulfoxide (DMSO; Sigma), control experiments were performed in the presence of equivalent concentrations of DMSO.

### Cell isolation and culture

RASMCs were prepared as previously described with minor modifications [19]. Briefly, RASMCs were isolated from the thoracic medial layer of adult male Wistar rat (180-200 g) by an enzymatic digestion with collagenase type 2 (60 U/mL; Worthington Biochemical Corporation, Lakewood, NJ, USA) and elastase (0.3 mg/mL; MP Biomedicals, Solon, Ohio, USA) for 3 hours at 37°C, with continuous slow shaking. After periods of 30 minutes, the suspension was centrifuged at 1300 rpm for 3 minutes, and the cells were collected and placed in Dulbecco's Modified Eagle Medium (DMEM; GIBCO, Invitrogen, Cergy Pontoise, France) containing antibiotics/antimycotic (100 U/mL penicillin, 100 µg/mL streptomycine, 0.25 µg/mL amphotericin B; GIBCO) and supplemented with 20% Fetal Bovine Serum “Gold” (FBS; PAA Laboratories, Les Mureaux, France). Cells obtained during the first period were discarded. Those obtained in the subsequent cycles were pooled, centrifuged, suspended in DMEM containing 20% FBS, seeded in a flask coated with collagen I (rat tail, BD Biosciences, Le Pont de Claix, France) and maintained at 37°C in a 95% air-5% CO_2_ humidified atmosphere. The medium was changed every 2 days with DMEM containing 10% FBS. At confluence, cells were detached with 0.05% trypsin containing 0.53 mM EDTA (GIBCO) and seeded in coated flasks (passage 1). All experiments were performed on cells cultured between passages 2 and 6. Cells were plated on collagen-coated Petri dishes for RT-PCR experiments and PDE activity assay or on collagen-coated glass coverslips for FRET experiments, at a density of 3.10^3^ or 10^4^ cells/cm^2^ for experiments performed at 24 or 48 hours, respectively. As shown in **Figure** **S1**, markers characteristic of cells with a synthetic phenotype were expressed in cultured RASMCs.

### Quantitative RT-PCR

Cells, maintained in DMEM containing 10% FBS for 48 hours after plating, were washed twice with cold PBS, immediately frozen and kept at −80°C. Cells were scrapped and homogenized using a tissue homogenizer (Bertin Technologies) in ice-cold TRI reagent (Molecular Research center, Cincinnati, USA). Total RNA was extracted using standard procedure. cDNA was synthesized from 1 µg total RNA using iSCRIPT cDNA synthesized (Biorad, Marnes-la-Coquette, France). Negative controls were performed without the reverse transcriptase. Then, qPCR was performed using the SYBR®-Green method on a CFX96 real-time PCR detection system (Biorad). Reactions were carried out in SYBR-Green master mix with 12.5 ng cDNA and 0.5 µM sense and anti-sense primers for subtypes of PDE 1 to 8 and housekeeping genes (TBP: Tata Box Binding Protein, RPL32: Ribosomal protein L32 and Ywhaz: 14–3–3 protein zeta/delta) (**Table** **1**). Negative controls were performed without cDNA template to check for exogenous contamination. The cycling conditions were 30 seconds at 95°C, following by 35 cycles with one step at 95°C for 5 seconds and another step at 60–62°C for 20 seconds. For each target gene, a standard curve was constructed from the analysis of serial dilution of cDNA and was used to determine efficiency (E). Threshold cycle (Ct) for target was subtracted from the geometric mean of Ct for housekeeping genes to calculate (1+E)^ΔCt^ according to the 2^ΔCt^ method. PCR products were analyzed by electrophoresis on a 3% agarose gel and visualized with GelRed® (Biotium, Hayward, USA) under UV light.

**Table 1:** Primers pairs used in RT-PCR to quantify mRNA expression.

Target	Accession number	Primers Forward 5′→ 3′ Reverse 5′→ 3′	Product size (Bp)	Annealing temperature (°C)
**PDE1A**	**NM_030871**	GAAGTTTCGCAGCATTGTCC GCAGGATATGTCAAACCAACC	**96**	**60**
**PDE1B**	**NM_022710**	TGCAGTCCACAACTGTCTCA CCCGGTTCAAAGAGAAGACA	**65**	**62**
**PDE1C**	**NM_031078**	GCAGTGCAAGCTGGGATATT TTACAGCCGGTGGATAGCTC	**82**	**60**
**PDE2A**	**NM_001143847**	CTGTGCTGGCTGCACTCTAC GAGGATAGCAATGGCCTGAG	**77**	**62**
**PDE3A**	**NM_017337**	ACCTCCCTGCCCTGCATAC CCTCTCTTGTGGTCCCATTC	**65**	**60**
**PDE3B**	**NM_017229**	GTGGCTACAAATGCACCTCA CTGGGCGAGAAAGATACAGA	**100**	**60**
**PDE4A**	**NM_ 013101**	CGTCAGTGCTGCGACAGTC CCAGCGTACTCCGACACACA	**190**	**60**
**PDE4B**	**NM_017031**	GATGAGCAGATCAGGGAACC GATGGGATTTCCACATCGTT	**81**	**60**
**PDE4C**	**XM_214325**	GACCCTGTCCTTCCTGTTGA AACCGTCTCAGGATCACACC	**99**	**60**
**PDE4D**	**NM_001113328**	GCCAGCCTTCGAACTGTAAG ATGGATGGTTGGTTGCACAT	**98**	**60**
**PDE7A**	**NM_031080**	TTGGAATTTTGATATCTTTCTGTTTG CTCAATCAATCCATGAAGACTAAA	**97**	**60**
**PDE7B**	**NM_080894**	GGCCATGCACTGTTACTTGA CCAGAGGTGTGAGGAAGCTC	**56**	**60**
**PDE8A**	**NM_198767**	CGGAGGTTTTCAGGAAATGA GGCCAACTGGCTTGAAGAT	**69**	**60**
**PDE8B**	**NM_199268**	GCCTCATTCGTTCAGACACA GGCGATTCTGTAGGCTTGG	**98**	**62**
**RPL32**	**NM_013226**	GCTGCTGATGTGCAACAAA GGGATTGGTGACTCTGATGG	**115**	**60**
**TBP**	**NM_001004198**	CGGTTTGCTGCAGTCATCAT GTGCACACCATTTTCCCAGA	**82**	**60**
**Ywhaz**	**NM_013011**	AGACGGAAGGTGCTGAGAAA GAAGCATTGGGGATCAAGAA	**127**	**60**

### Cyclic AMP-PDE activity assay

Cells, maintained in DMEM containing 10% FBS for 48 hours after plating, were washed twice with cold PBS, homogenized using a tissue homogenizer (Bertin Technologies) in ice-cold lysis buffer (containing: NaCl 150 mM, HEPES 20 mM, EDTA 2 mM, glycerol 10%, NP40 0.5% and microcystin 1 µM) and centrifuged at 12,000 g for 10 min at 4°C. Protein concentration was determined using the bicinchoninic acid protein assay, according to the manufacturer's protocol (Pierce, Thermo Fisher Scientific, Brebières, France). The cAMP-PDE activity was measured in the supernatant according to a modification of the two-step assay procedure method described by Thompson and Appleman [20], using 20–30 µg of RASMCs proteins in a total volume of 200 µl including 10 mM Tris-HCl, pH 8.0, 10 mM MgCl_2_, 5 mM β-mercaptoethanol and 1 µM cAMP and supplemented with 10^5^ cpm [^3^H]-cAMP, as detailed previously [21]. To evaluate PDE families-specific activities, the assay was performed in the absence or presence of either one or a combination of several selective PDE inhibitors: 10 or 50 µM MIMX for PDE1, 100 nM BAY for PDE2, 1 µM Cil for PDE3, 10 µM Ro for PDE4, 50 µM BRL for PDE7 and 1 mM IBMX as a non-selective PDE inhibitor. The residual hydrolytic activity observed in the presence of PDE inhibitors was expressed as a percentage of the total cAMP-PDE activity, corresponding to the cAMP-PDE activity in the absence of inhibitor.

### FRET imaging

Two different FRET-based cAMP sensors were used: Epac1-camps, containing the single cAMP-binding domain of Epac1 fused to an enhanced yellow fluorescent protein (YFP) and an enhanced cyan fluorescent protein (CFP) [13]; and the plasma membrane-targeted cAMP sensor Epac2-camps, called pm-Epac2-camps, corresponding to Epac2-camps (containing the cAMP-binding domain B of Epac2 fused to YFP and CFP) which was N-terminally-modified with the ″SH4″ motif of Lyn kinase [22]. FRET between CFP and YFP appears in the absence of cAMP. Upon increase in cAMP concentration, cAMP binding to its Epac domain promotes reversible conformational changes of the sensor, resulting in a decrease in FRET between CFP and YFP [13].

RASMCs were transfected with a pcDNA3 plasmid encoding Epac1-camps by using Lipofectamine^TM^ 2000 (Invitrogen) diluted in Opti-MEM medium® (Invitrogen) according to the manufacturer's instructions. After 6 hours, the transfection medium was replaced with DMEM containing 10% FBS. In some experiments, the RASMCs were infected with an adenovirus encoding pm-Epac2-camps (MOI 600 pfu/cell) in DMEM containing 10% FBS. FRET experiments were performed 42 hours after transfection with Epac1-camps or 24 hours after infection with pm-Epac2-camps. We previously determined these incubation times to get an optimal subcellular localization of the sensors.

Cells were maintained in a K^+^-Ringer solution containing (in mM): NaCl 121.6, KCl 5.4, MgCl_2_ 1.8, CaCl_2_ 1.8, NaHCO_3_ 4, NaH_2_PO_4_ 0.8, D-glucose 5, sodium pyruvate 5, HEPES 10 (pH 7.4), at room temperature. To study changes in FRET signals in response to pharmacological agents, the cell of interest was continuously and locally perfused with K^+^-Ringer solutions containing or not these agents, using a microperfusion system allowing rapid applications of these solutions. Iso was applied either at steady state to perform cumulative concentration-response curves or as a pulse of 15 s (at a submaximal concentration) to evaluate the effect of pharmacological agents on the dynamics of its response. PDE inhibitors and β-AR antagonists were applied 3 min before the brief application of Iso and maintained throughout the experiment.

Images were captured every 5 s using the 40X oil immersion objective of an inverted microscope (Nikon TE 300) connected to a software-controlled (Metafluor, Molecular Devices) cooled charge-coupled device camera (Sensicam PE; PCO, Kelheim, Germany). CFP was excited during 150–300 ms by a Xenon lamp (100 W, Nikon, Champigny-sur-Marne, France) using a 440/20BP filter and a 455LP dichroic mirror. Dual emission imaging of CFP and YFP was performed using an Optosplit II emission splitter (Cairn Research, Faversham, UK) equipped with a 495LP dichroic mirror and BP filters 470/30 and 530/30, respectively. Average fluorescence intensity was measured in a region of interest comprising the entire cell. For analysis, background was subtracted and YFP intensity was corrected for CFP spillover into the 535 nm channel before calculating the ratio of CFP/YFP emitted fluorescence intensities. Data were expressed as a percentage of the CFP/YFP ratio measured before application of the drug. Ratio images were obtained with ImageJ software (National Institutes of Health). Kinetic parameters of the Iso-induced FRET signal (t_max_: time to peak, t_1/2_on: time to half-peak, t_1/2_off: time from the peak to obtain half recovery) were determined using Microsoft Excel software. EC_50_ value (concentration that produces 50% of the maximum response) was estimated in each individual concentration-response curves using the Boltzman equation fit using Origin 6 software.

### Data analysis and statistics

Data are represented as mean ± SEM of *N* experiments in RT-PCR studies and cAMP-PDE activity assay or *n* cells in FRET imaging experiments. The different parameters were compared using Student's *t*-test for paired or unpaired data, where appropriate. A difference was considered statistically significant when P<0.05.

## Results

### Expression pattern of PDE isoforms in cultured RASMCs

**Figure 1:**
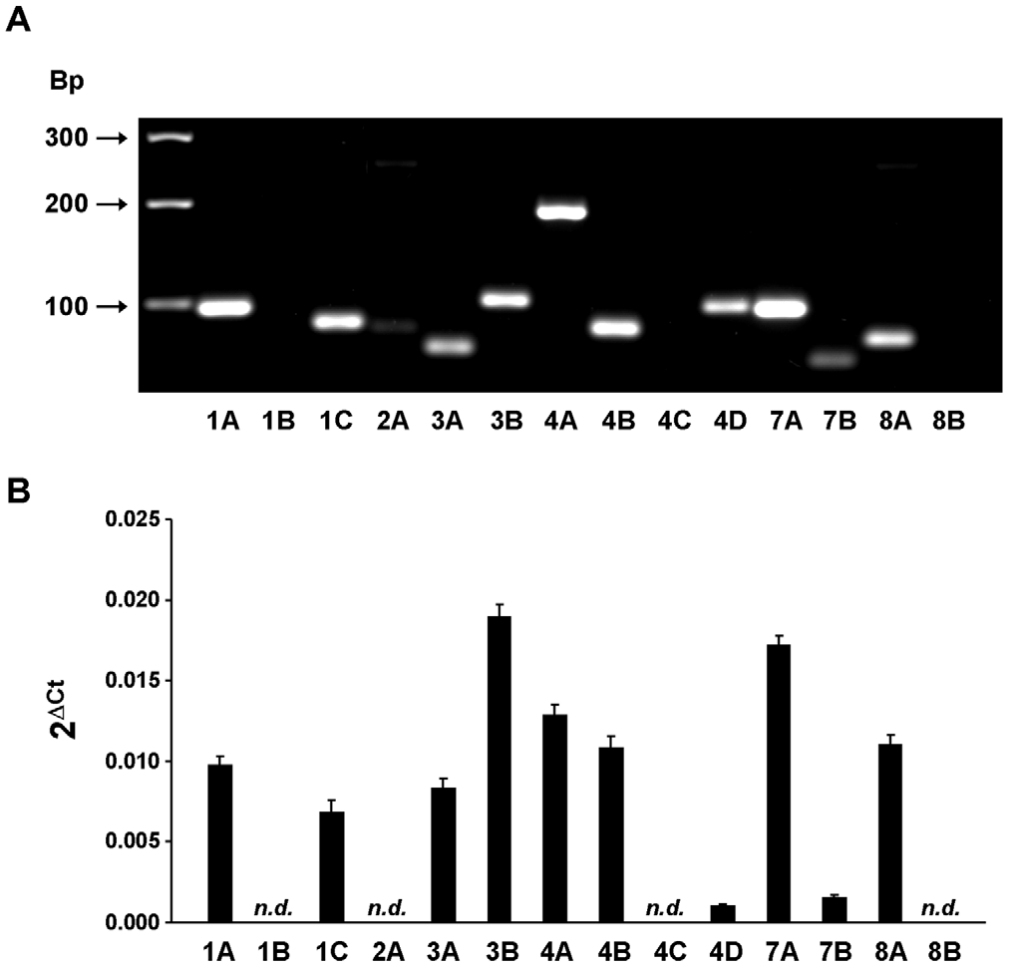
Expression analysis of mRNA encoding cAMP-PDE isoforms in RASMCs. RT-qPCR reactions were carried out on mRNAs isolated from RASMCs. The expression of PDE1A (1A), PDE1B (1B), PDE1C (1C), PDE2A (2A), PDE3A (3A), PDE3B (3B), PDE4A (4A), PDE4B (4B), PDE4C (4C), PDE4D (4D), PDE7A (7A), PDE7B (7B), PDE8A (8A) and PDE8B (8B) was analyzed. ***A***: PCR products were resolved by electrophoresis on a 3% agarose gel. Shown is the picture of a representative gel stained with GelRed®. Position of molecular weight markers is indicated in base pairs (Bp). ***B***: mRNA expression was expressed using the 2^ΔCt^ method as described in Materials and methods. Data are mean±SEM of 9 experiments. n.d. not detectable.

mRNA species encoding PDEs 1A, 1C, 3A, 3B, 4A, 4B, 4D, 7A, 7B and 8A were detected in cultured RASMCs (**Figure** **1**). RT-PCR products for PDE 1B, 2A, 4C and 8B isoforms were absent.

### cAMP-PDE activity in cultured RASMCs

**Figure 2:**
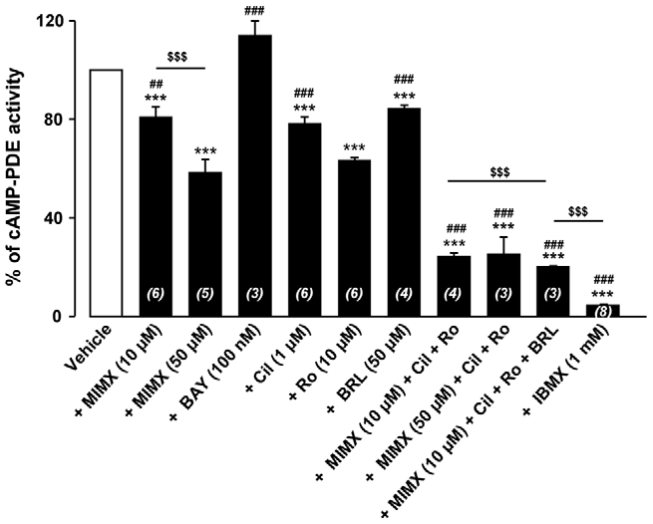
cAMP-PDE activity in RASMCs. cAMP-PDE activity was determined in lysates of cultured RAMSCs in the absence (*vehicle*) or presence of selective PDE inhibitors (PDE1: 10 or 50 µM MIMX; PDE2: 100 nM BAY; PDE3: 1 µM Cil; PDE4: 10 µM Ro; PDE7: 50 µM BRL) or a non-selective PDE inhibitor (1 mM IBMX) or a combination of several inhibitors as indicated. Results are expressed in % of cAMP-PDE activity measured in the absence of inhibitors (*vehicle*). Data are mean±SEM of 3–6 independent experiments. *** P<0.001 *versus* vehicle;^ ##^ P<0.01 and ^###^ P<0.001 *versus* Ro; ^$$$^ P<0.001 as indicated.

Total cAMP-PDE activity measured in lysates from cultured RASMCs was 37.4±7.1 pmol/min/mg protein (n = 6). To determine which PDE families contribute to this total hydrolyzing activity, the assay was also conducted in the presence of different PDE inhibitors (**Figure** **2**). Consistent with the absence of PDE2 mRNA expression, BAY (100 nM), a selective PDE2 inhibitor [23], had no effect on cAMP-PDE activity. Cil (1 µM, a selective PDE3 inhibitor [24]) and Ro (10 µM, a selective PDE4 inhibitor [25]) reduced cAMP-PDE activity by 20% and 40%, respectively. As no perfect PDE1 inhibitor is commercially available, we used MIMX which blocks PDE1 activity by interfering with its catalytic site with a low micromolar affinity and exhibits a selectivity over other PDEs of 30- to 50-fold [25]-[27]. MIMX decreased cAMP-PDE activity by 22% and 37%, when used at 10 µM and 50 µM, respectively. In the simultaneous presence of MIMX (10 µM), Cil and Ro, total cAMP-PDE activity was reduced by 75%. Increasing the concentration of MIMX to 50 µM had no further effect under this condition. The PDE7 inhibitor BRL (50 µM) [28] lowered the total cAMP-PDE activity by 16%. However, BRL had no additive inhibitory effect on the cAMP-PDE activity measured in the presence of MIMX (10 µM), Cil and Ro which questions its specificity on PDE7. Finally, the broad-spectrum PDE inhibitor IBMX (1 mM) [25] inhibited the total cAMP-PDE activity by 96%. In summary, the rank order of PDE families contributing to global cAMP-PDE activity in cultured RASMCs was PDE4> PDE3 = PDE1. PDE2 activity is absent and PDE7 activity uncertain.

### FRET measurements of cytosolic cAMP signals in cultured RASMCs

**Figure 3:**
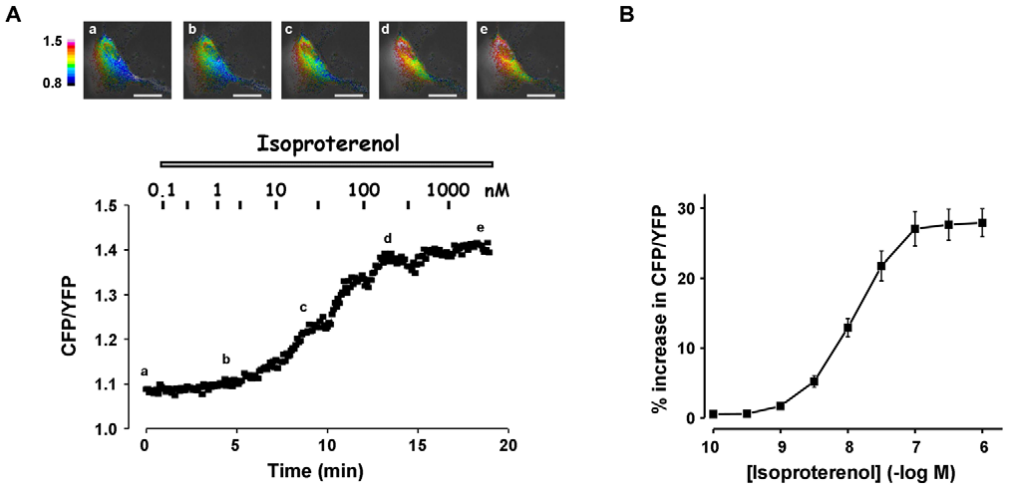
Effect of steady-state activation of β-AR on cytosolic [cAMP] in RASMCs. Cytosolic cAMP measurements were conducted using the FRET-based cAMP sensor Epac1-camps in cultured RAMSCs cells incubated with cumulative increasing concentrations of isoproterenol (0.1 nM to 1 µM). ***A***: Representative cAMP signals monitored in one cell. Pseudocolor images reflecting the CFP/YFP ratio were recorded at the times indicated by the letters on the graph. ***B***: Concentration-response curve of isoproterenol effect as shown in *A*. Data are mean±SEM of 7 independent cells.

**Figure 4:**
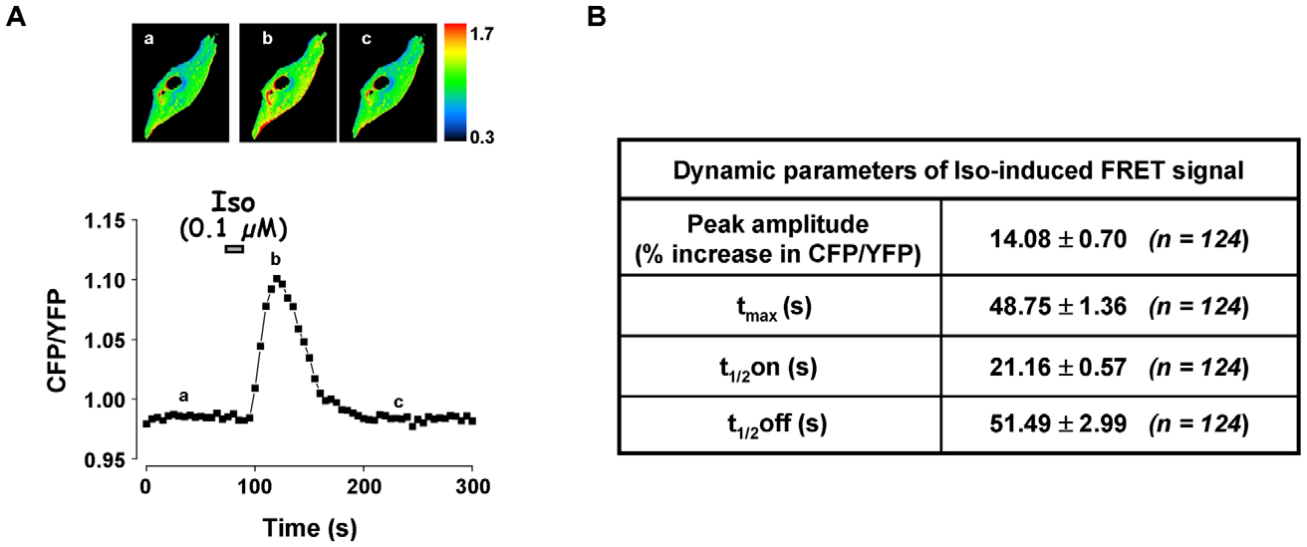
Effect of transient activation of β-AR on cytosolic [cAMP] in RASMCs. Cytosolic cAMP measurements were conducted in cultured RAMSCs cells using the FRET-based cAMP sensor Epac1-camps in response to a short application of isoproterenol (Iso, 0.1 µM, 15 s). ***A***: Representative cAMP signals monitored in one cell. Pseudocolor images reflecting the CFP/YFP ratio were recorded at the times indicated by the letters on the graph. ***B***: Dynamic parameters (peak amplitude, t_max_, t_1/2_on, and t_1/2_off) of isoproterenol-induced FRET signal as shown in *A*. Data are mean±SEM of 124 independent cells.

We then evaluated the functional contribution of the different PDE isoforms in controlling intracellular cAMP concentration ([cAMP]_i_) in RASMCs. [cAMP]_i_ changes in response to Iso were monitored in real-time by fluorescence imaging using the FRET-based cAMP sensor Epac1-camps. As illustrated on the images in **Figure** **3A** and **Figure** **4A**, the CFP/YFP ratio fluorescence was distributed throughout the cytosol in cells expressing Epac1-camps, indicating a cytosolic localization of the probe. Cumulative increasing concentrations of Iso (0.1 nM to 1 µM) induced a significant increase in CFP/YFP ratio in a concentration-dependent manner, with a maximum response of 27.9±2.0% and an EC_50_ value of 18.1±2.2 nM (n = 7) (**Figure** **3B**). These alterations of FRET signal reflect the production of cytosolic cAMP upon β-AR stimulation. To analyze the dynamics of this cAMP signal, Iso was then applied transiently at the concentration of 0.1 µM. As shown in **Figure** **4**, a 15 s-pulse of Iso induced a rapid increase in the CFP/YFP ratio to reach a maximum of 14.1±0.7% at 48.7±1.4 s (n = 124) before returning to baseline with a t_1/2_off of 51.5±3.0 s. Thus, a short β-AR stimulation induced a transient increase in cytosolic [cAMP]_i_, suggesting that cAMP is rapidly metabolized in RASMCs, most likely through cAMP hydrolysis by PDEs.

### Role of PDE families in the control of cytosolic cAMP concentrations in cultured RASMCs

**Figure 5:**
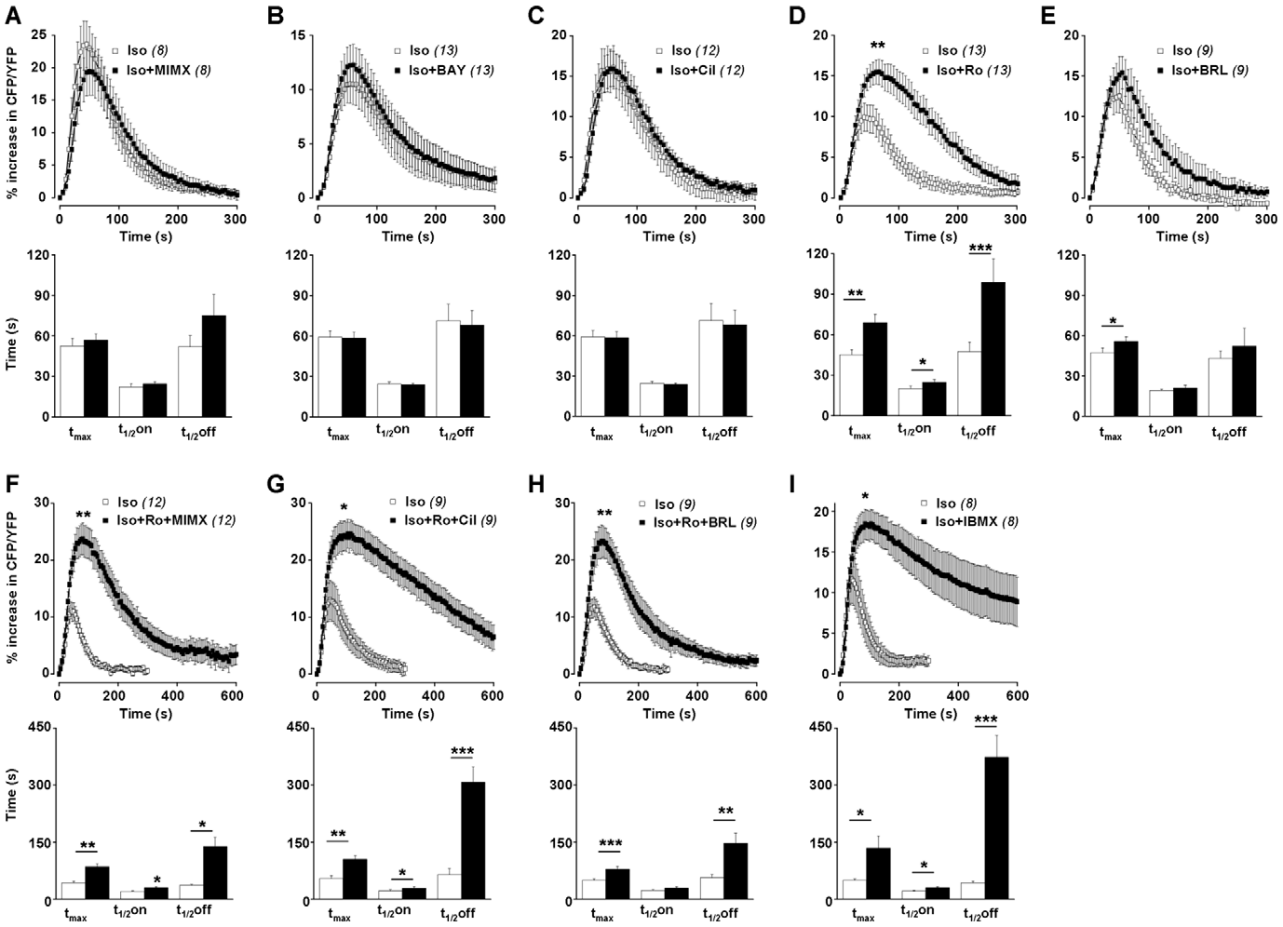
Effect of PDE inhibition on β-AR-induced cytosolic cAMP signal in RASMCs. Cytosolic cAMP measurements were conducted in cultured RAMSCs cells using the FRET-based cAMP sensor Epac1-camps in response to a short application of isoproterenol (Iso, 0.1 µM, 15 s) after a pre-treatment in the absence (□) or presence (▪) of one selective PDE family inhibitor (***A***: 50 µM MIMX for PDE1; ***B***: 100 nM BAY for PDE2; ***C***: 1 µM Cil for PDE3; ***D***: 10 µM Ro for PDE4; ***E***: 50 µM BRL for PDE7) or a combination of several inhibitors (***F***: 10 µM Ro + 50 µM MIMX +; ***G***: 10 µM Ro + 1 µM Cil; ***H***: 10 µM Ro + 50 µM BRL) or the non-selective PDE inhibitor (***I***: 100 µM IBMX). *Top* and *lower panels* represent the mean variation of CFP/YFP ratio and the corresponding kinetic parameters, respectively. Data are mean±SEM of 8–13 independent cells as indicated. * P<0.05, ** P<0.01, *** P<0.001 *versus* Iso.

**Figure 6:**
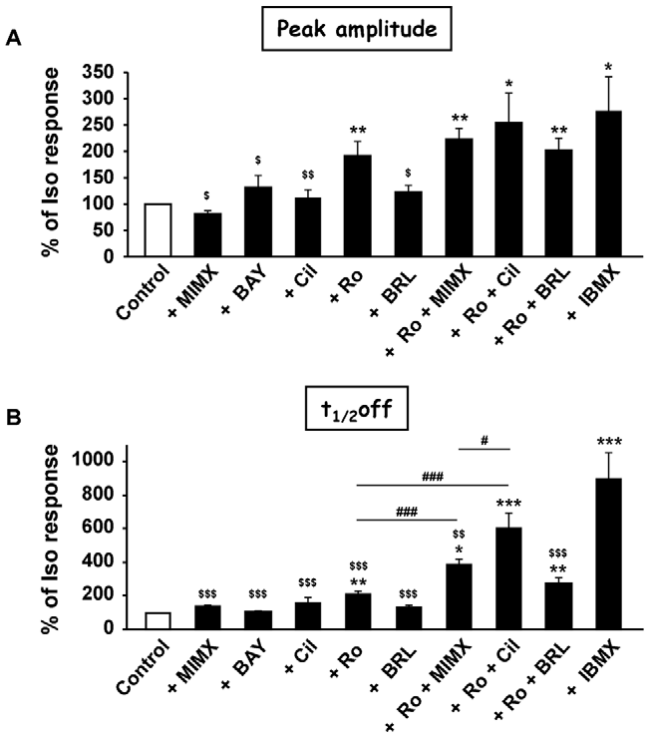
Effect of PDE inhibitors on the peak amplitude and t_1/2_off of β-AR-induced cytosolic cAMP signal in RASMCs. Dynamic parameters (peak amplitude, ***A*** and t_1/2_off, ***B***) obtained in Figure 5 are expressed in % of isoproterenol-induced response (*Control*). Data are mean±SEM of 8–13 independent cells as indicated. * P<0.05, ** P<0.01, *** P<0.001 *versus* Control; ^$^ P<0.05, ^$$^ P<0.01 and ^$$$^ P<0.001 *versus* IBMX; ^#^ P<0.05, ^###^ P<0.001 as indicated.

To evaluate the functional contribution of the different PDE families in regulating the cAMP response to β-AR stimulation, RASMCs were incubated in the presence of PDE inhibitors during 3 min before Iso pulse. Application of PDE inhibitors alone, with the exception of BAY and BRL, induced a slight but significant increase in basal FRET ratio, which was maximal with 50 µM MIMX (the PDE1 inhibitor) and 100 µM IBMX (the non-selective PDE inhibitor) (**Table** **2**). 50 µM MIMX (**Figure** **5A**), 100 nM BAY (the PDE2 inhibitor, **Figure** **5B**) and 1 µM Cil (the PDE3 inhibitor, **Figure** **5C**) had no effect on the dynamics of the Iso-induced FRET signal. 50 µM BRL (the PDE7 inhibitor, **Figure** **5E**) had only minor effects on its kinetics (with a slight increase in the time to peak of 17%, n = 9, P<0.05), whereas 10 µM Ro (the PDE4 inhibitor, **Figure** **5D**) markedly increased its amplitude (by 52%, n = 13, P<0.01) and significantly delayed its onset and recovery phases. IBMX (**Figure** **5I**) also increased the amplitude of Iso response (by 76%, n = 9, P<0.05) and dramatically prolonged its duration. The IBMX effect on the decay phase was significantly higher than that induced by Ro (t_1/2_off increased by 99% and 770% with Ro and IBMX, respectively, P<0.001; **Figure** **6B**). This indicates that, in RASMCs, PDE4 is an essential regulator of β-AR-elicited cytosolic cAMP signals, but that other PDEs might also contribute to this regulation.

We then evaluated the effect of the simultaneous inhibition of PDE4 and one of the 3 other PDEs detected in these cells (as shown by PDE activity assay: PDE1, PDE3 and also PDE7) on the Iso response. Ro+MIMX significantly increased the amplitude of Iso response (by 109%, n = 12) and delayed its onset and recovery phases (**Figure** **5F**). Only the effect of Ro+MIMX on the decay phase was significantly higher than that induced by Ro alone [t_1/2_off increased by 99% (n = 13) and 280% (n = 12) with Ro and Ro+MIMX, respectively, P<0.001; **Figure** **6**]. However, this effect was still lower than that induced by IBMX. It should be noticed that a similar potentiating effect of MIMX in the presence of Ro was also observed when MIMX was used at a smaller concentration, 10 µM (**Figure** **S2**). Ro+Cil significantly increased the amplitude of Iso response (by 80%, n = 9) and drastically prolonged its duration (**Figure** **5G**), with an effect on the decay phase which was significantly higher than that induced by Ro alone [t_1/2_off increased by 99% (n = 13) and 377% (n = 9) with Ro and Ro+Cil, respectively, P<0.001; **Figure** **6B**] and by Ro+MIMX [t_1/2_off increased by 280% (n = 12) and 377% (n = 9) with Ro+MIMX and Ro+Cil, respectively, P<0.001; **Figure** **6B**]. In the presence of Ro+BRL (**Figure** **5H**), the Iso response was significantly increased in amplitude (by 90%, n = 9) and duration (t_1/2_off increased by 160%), but to a similar extent to that obtained in the presence of Ro alone (**Figure** **6**). Altogether, these results suggest that PDE4 inhibition unmasks the effect of PDE3 and PDE1 on cytosolic cAMP generated by β-AR stimulation.

**Table 2:** Effect of PDE inhibition on basal cytosolic cAMP signal in RASMCs.

PDE inhibitor	% increase in CFP/YFP
Vehicle	0.8±0.1 (n = 9)
MIMX (50 µM)	3.1±0.3 (n = 8) ***
BAY (100 nM)	0.3±0.1 (n = 13)
Cil (1 µM)	1.7±0.4 (n = 12) *
Ro (10 µM)	1.8±0.4 (n = 13) *
BRL (50 µM)	1.4±0.3 (n = 9)
Ro (10 µM) + MIMX (50 µM)	2.8±0.3 (n = 12) ***^, #^
Ro (10 µM) + Cil (1 µM)	2.4±0.7 (n = 9) *
Ro (10 µM) + BRL (50 µM)	1.8±0.2 (n = 9) **
IBMX (100 µM)	3.4±1.1 (n = 8) *

Cytosolic cAMP measurements were conducted in cultured RAMSCs cells using the FRET-based cAMP sensor Epac1-camps. The % increase in CFP/YFP ratio induced by the different PDE inhibitors or the vehicle was calculated after a 3 min-incubation. Data are mean±SEM of 8–13 independent cells as indicated. * P<0.05, ** P<0.01, *** P<0.001 *versus* vehicle; ^#^ P<0.05 *versus* Ro.

### Role of β_1_- and β_2_-AR subtypes in cytosolic cAMP production in cultured RASMCs

**Figure 7:**
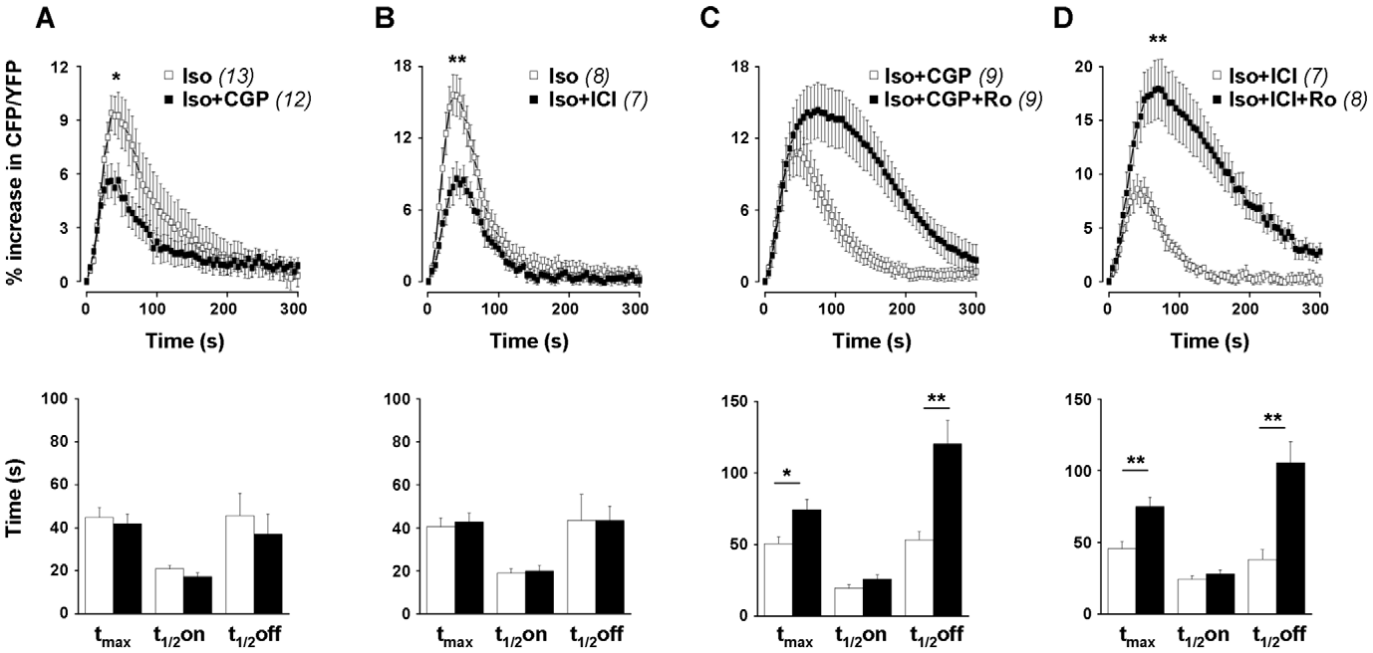
Effect of β_1_-AR and β_2_-AR antagonists on β-AR-induced cytosolic cAMP signals in RASMCs. Cytosolic cAMP measurements were conducted in cultured RAMSCs cells using the FRET-based cAMP sensor Epac1-camps in response to a short application of isoproterenol (Iso, 0.1 µM, 15 s) after a pre-treatment in the absence or presence of β-AR antagonists and PDE4 inhibitor. ***A, B***: Effect of β_1_-AR (100 nM CGP-20712A, *CGP*; ***A***) and β_2_-AR (5 nM ICI 118,551, *ICI*; ***B***) antagonists on Iso response. ***C, D***: Effect of PDE4 inhibitor (10 µM Ro) on Iso response obtained in the presence of β_1_-AR (*Iso+CGP*; ***C***) or β_2_-AR (*Iso+ICI*; ***D***) antagonists. *Top* and *lower panels* represent the mean variation of CFP/YFP ratio and the corresponding kinetic parameters, respectively. Data are mean±SEM of 7-13 independent cells as indicated. * P<0.05, ** P<0.01 *versus* Iso (*A, B*), Iso+CGP (*C*) or Iso+ICI (*D*).

To evaluate the contribution of β_1_- and β_2_-AR subtypes in cytosolic cAMP production elicited by Iso, RASMCs were incubated in the presence of β_1_- or β_2_-AR antagonists during 3 min before the Iso pulse. The selective β_1_-AR antagonist CGP-20712A was used at a concentration of 100 nM, which is about 100-fold higher than its binding affinity [29], [30]. At this concentration, CGP-20712A did not modify the FRET signal induced by a selective β_2_-AR agonist (**Figure** **S3**), confirming its selectivity towards β_1_-ARs in our experimental conditions. By contrast, CGP-20712A significantly decreased the amplitude of Iso response by 39% without affecting its kinetics (**Figure** **7A**), indicating that β_1_-ARs are involved in this response. To antagonize β_2_-ARs, we used ICI 118,551, which exhibits a binding affinity for β_2_-ARs of around 1 nM and a selectivity ratio for β_2_-ARs over β_1_-ARs of about 70 [30]. We evaluated the effect of different concentrations of ICI 118,551 (1, 5, 10 and 100 nM) on FRET signal induced by Iso in RASMCs. ICI 118,551 significantly reduced the amplitude of Iso response in a concentration-dependent manner [by 14% (n = 6), 48% (n = 7), 55% (n = 5) and 90% (n = 9) at 1, 5, 10 and 100 nM, respectively], without affecting its kinetics (**Figure** **S4** and **Figure** **7B** for ICI 118,551 at 5 nM), indicating that β_2_-ARs are involved in this response. In the following experiments, we decided to use ICI 118,551 at 5 nM, a concentration which should preserve its selectivity towards β_2_-ARs according to its binding affinities.

As PDE4 is the main PDE family controlling Iso-induced cytosolic cAMP signals, we then evaluated the role of PDE4 in hydrolyzing cAMP produced by either β_1_- or β_2_-AR stimulation. The β_1_-AR response was elicited through an Iso pulse in the presence of the β_2_-AR antagonist ICI 118,551, whereas the β_2_-AR response was elicited through an Iso pulse in the presence of the β_1_-AR antagonist CGP-20712A. The PDE4 inhibitor (10 µM Ro) slightly but not significantly increased the amplitude of the Iso+CGP-20712A response, and significantly prolonged its duration with a strong effect on its recovery phase (t_1/2_off increased by 155%, n = 9, P<0.01; **Figure** **7C**). Similarly, the Iso+ICI 118,551 response (**Figure** **7D**) was markedly increased by Ro in amplitude (by 120%, n = 7–8, P<0.01) and duration (t_1/2_off increased by 178%, n = 9, P<0.01). This indicates that PDE4 hydrolyses cytosolic cAMP pool generated by both β_1_- and β_2_-AR stimulations.

### Role of PDE families in the control of submembrane cAMP concentrations in cultured RASMCs

**Figure 8:**
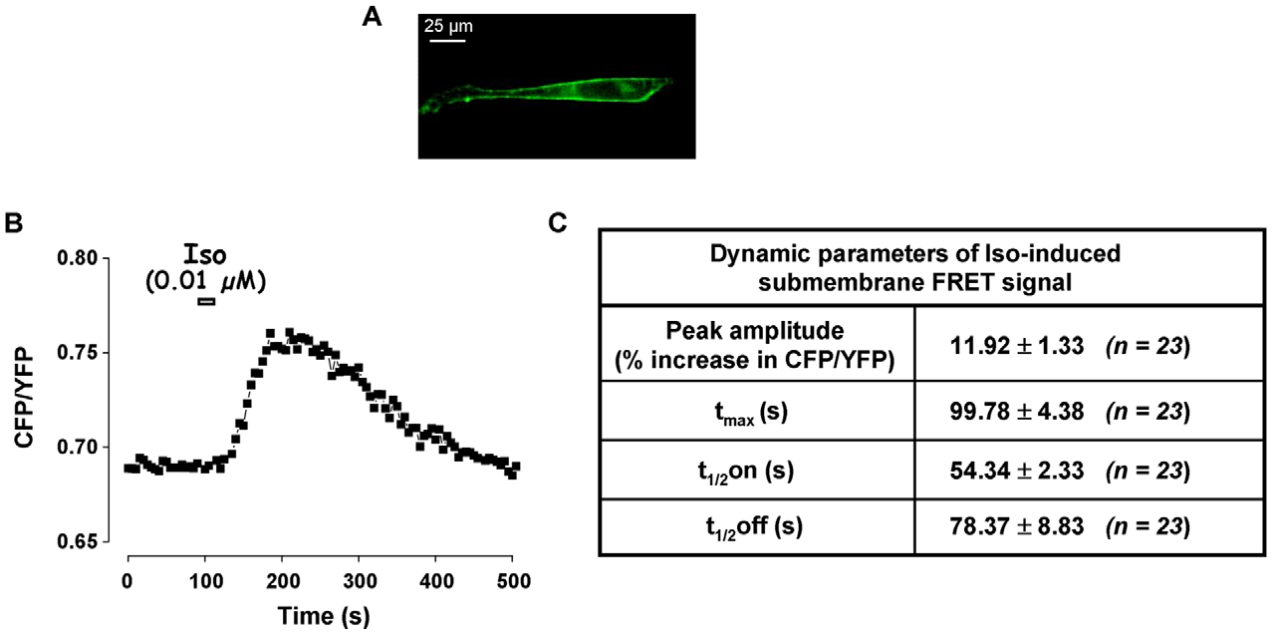
Effect of transient activation of β-AR on submembrane [cAMP] in RASMCs. Submembrane cAMP measurements were conducted in cultured RASMCs cells using the plasma membrane-targeted FRET-based cAMP sensor pm-Epac2-camps in response to a short application of isoproterenol (Iso, 0.01 µM, 15 s). ***A***: Submembrane localization of pm-Epac2-camps was ascertained by recording the CFP emission. ***B***: Variation of the CFP/YFP ratio monitored in one cell. ***C***: Dynamic parameters (peak amplitude, t_max_, t_1/2_on, and t_1/2_off) of isoproterenol-induced FRET signal as shown in *B*. Data are mean±SEM of 23 independent cells.

**Figure 9:**
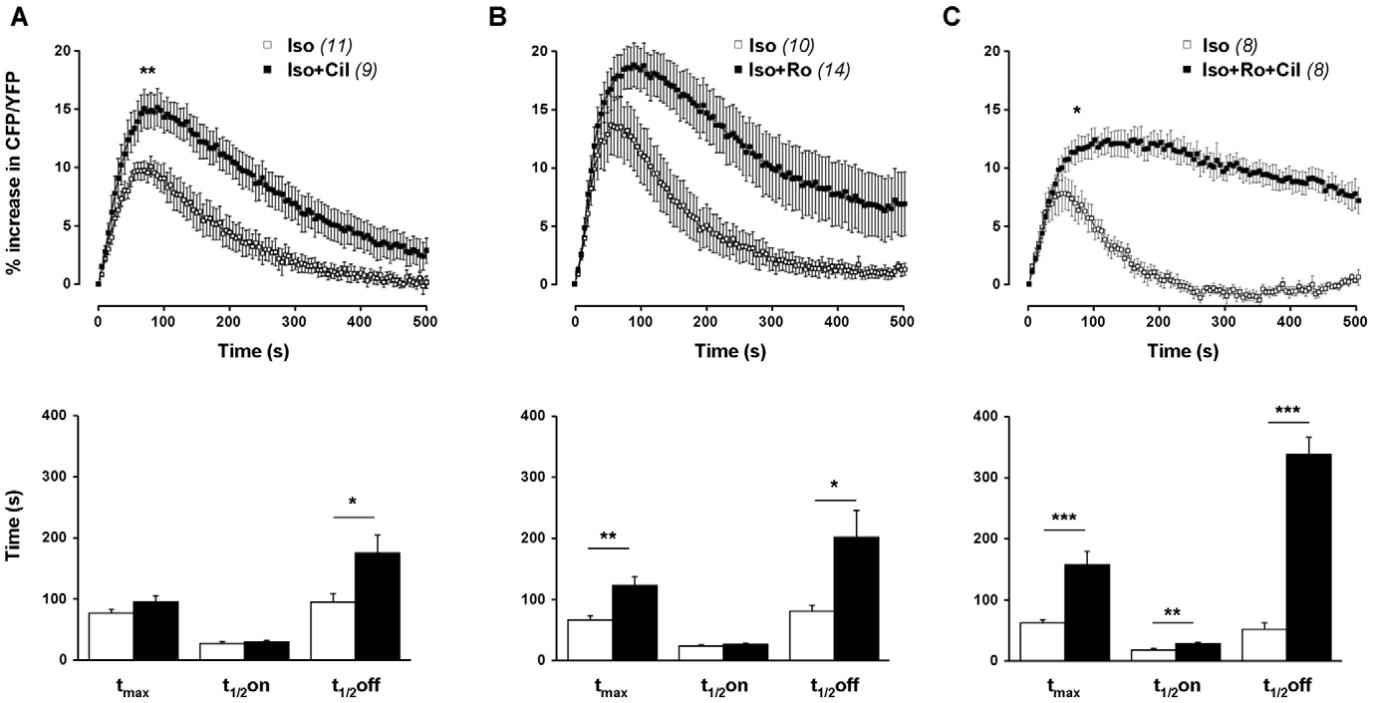
Effect of PDE inhibition on β-AR-induced submembrane cAMP signal in RASMCs. Submembrane cAMP measurements were conducted in cultured RASMCs cells using the FRET-based cAMP sensor pmEpac2-camps in response to a short application of isoproterenol (Iso, 0.01 µM, 15 s) after a pre-treatment in the absence (□) or presence (▪) of one selective PDE family inhibitor (***A***: 1 µM Cil for PDE3; ***B***: 10 µM Ro for PDE4) or a combination of 10 µM Ro + 1 µM Cil (***C***). *Top* and *lower panels* represent the mean variation of CFP/YFP ratio and the corresponding kinetic parameters, respectively. Data are mean ± SEM of 8-14 independent cells as indicated. * P<0.05, ** P<0.01, *** P<0.001 *versus* Iso.

To directly monitor cAMP dynamics in the submembrane compartment, we used the modified FRET-based cAMP sensor, pm-Epac2-camps, which was effectively targeted to the plasma membrane (**Figure** **8A**). As shown in **Figure** **8B**, pm-Epac2-camps generated a FRET response upon transient stimulation with Iso at the concentration of 10 nM. The FRET signal reached a maximum of 11.9±1.3% at 99.8±4.4 s (n = 23 cells) before returning to baseline with a t_1/2_off of 78.4±8.8 s (**Figure** **8C**). We then investigated the role of PDE3 and PDE4 families in regulating submembrane cAMP produced by β-AR stimulation. Application of 1 µM Cil or 10 µM Ro slightly increased the basal FRET ratio (**Table** **3**). Both PDE3 and PDE4 inhibitors significantly affected the Iso-induced FRET signal by increasing its amplitude (by 51% and 38%, respectively) and delaying its recovery phase (**Figure** **9A and B**). When Cil and Ro were applied concomitantly, the basal FRET ratio was markedly enhanced (**Table** **3**) and the Iso response was significantly increased in amplitude by 59% (P<0.05, n = 8) and dramatically prolonged in duration (t_1/2_off increased by 548%, compared to 150% and 85% with Cil and Ro alone, respectively, P<0.001; **Figure** **9C**). It should be noted that the effect of PDE inhibitors on the amplitude of Iso response was underestimated here given their effect on the basal FRET ratio. Altogether, these results suggest that, unlike in the cytosol, both PDE3 and PDE4 control submembrane cAMP concentration in RASMCs.

**Table 3:** Effect of PDE inhibition on basal submembrane cAMP signal in RASMCs.

PDE inhibitor	% increase in CFP/YFP
Vehicle	0.8±0.1 (n = 10)
Cil (1 µM)	1.8±0.5 (n = 9) ***
Ro (10 µM)	2.6±0.3 (n = 14) ***
Ro (10 µM) + Cil (1 µM)	5.2±0.9 (n = 8) ***^, ##^

Submembrane cAMP measurements were conducted in cultured RASMCs cells using the FRET-based cAMP sensor pm-Epac2-camps. The % increase in CFP/YFP ratio induced by the different PDE inhibitors or the vehicle was calculated after a 3 min-incubation. Data are mean±SEM of 8–14 independent cells as indicated. *** P<0.001 *versus* vehicle; ^##^ P<0.01 *versus* Cil and *versus* Ro.

## Discussion

In this study, we evaluated the role of individual PDEs in regulating the spatiotemporal dynamics of cAMP signals in cultured RASMCs. The main findings are as follows: (1) mRNAs encoding several cAMP-PDE isoforms (including PDE7A, 7B, 8A) are present in cultured RASMCs; (2) the rank order of PDE activity in RASMCs is PDE4 > PDE1  =  PDE3; (3) Iso strongly increases cytosolic cAMP concentration, through both β_1_- and β_2_-AR activation; (4) PDE4 is the main PDE controlling the cytosolic cAMP signal elicited by β-AR stimulation. However, PDE4 inhibition unmasks an effect of PDE3 and PDE1 in hydrolyzing cytosolic cAMP; (5) both PDE3 and PDE4 contribute to regulate basal and β-AR-stimulated cAMP levels in the submembrane compartment.

Cell culture is known to induce a phenotypic switch of vascular smooth cells associated with modifications of PDE expression [16]–[18]. In this study, we performed a large PDE activity assay to characterize our cultured RASMC model in terms of biochemical cAMP-PDE activity. By using selective pharmacological inhibitors of PDE3 and PDE4 (cilostamide and Ro-20-1724, respectively) [24], [25], we observed that the most important cAMP-PDE was PDE4, accounting for around 40% of total cAMP hydrolyzing activity, whereas PDE3 accounted only for 20%. In this regard, mRNAs encoding 2 PDE3 isoforms (3A and 3B) and 3 PDE4 isoforms (4A, 4B and 4D) were detected in these cells. This is in accordance with Dunkerley *et al* who described a reduced PDE3/PDE4 activity ratio in cultured rat aorta cells compared to fresh cells; this modification appeared from the passage 1 of culture and was correlated with a reduced PDE3 activity [16]. Other classical cAMP-PDEs include PDE1 and PDE2. Here, we show that mRNAs encoding PDE1A and 1C but not 1B were expressed and that PDE1 accounted for at least 20% of total PDE activity according to the inhibitory effect of 10 µM MIMX. PDE1 protein expression was previously described in cultured aorta SMCs isolated from several species including rat [17], but PDE1 hydrolyzing cAMP activity was essentially shown in cells from other species [17], [31], [32]. Increasing MIMX concentration to 50 µM further increased the inhibitory effect of MIMX when used alone, but had no additive effect in the presence of PDE3 and PDE4 inhibitors suggesting that increasing MIMX concentration induces non-selective inhibition, probably towards PDE3 or PDE4, according to its affinity values [25], [27]. Finally, we confirmed the absence of PDE2 expression and activity in our conditions, as shown in previous works [33]. Thus, cultured RASMCs exhibited PDE1+PDE3+PDE4 activities, which contributed to 80% of total cAMP-PDE activity. However, IBMX-sensitive PDE activity represented about 96% of total activity. This suggests either that the concentrations of PDE inhibitors are not sufficient to fully block the related PDE activities, or that PDEs other than PDE1, 3 and 4 participate in the cAMP hydrolyzing PDE activity. Other IBMX-sensitive cAMP-PDEs include PDE7, PDE10 and PDE11. Phillips *et* *al* have shown that PDE7 mRNA is expressed in cultured RASMCs (at passage 2), and proposed that PDE7-PDE11 account for less than 10% of total cAMP-PDE activity of these cells [5]. Here, we observed that cultured RASMCs (at passages 2–6) express both PDE7A and PDE7B mRNAs, PDE7A being dominant. We further investigated the potential role of PDE7 in total cAMP-hydrolyzing activity by using a recently described PDE7 inhibitor (BRL) [28]. BRL decreased total cAMP-PDE activity by 16%, but had no additive effect to that induced by the association of PDE1+PDE3+PDE4 inhibitors. Furthermore, BRL did not alter the cAMP concentration measured by FRET-based imaging in intact living cells. Thus, the effect of BRL observed *in vitro* in cell lysates might be explained by a non-selective action on other PDEs than PDE7, particularly PDE4, for which BRL exhibits some affinity [28]. Therefore, the function of PDE7 isoforms in RASMCs remains to be elucidated by using more specific tools. In the absence of commercialized selective PDE10 and PDE11 inhibitors, we were unable to evaluate the contribution of these two isoforms in our PDE activity assay. To conclude, despite some limits of the pharmacological approach used in the PDE activity assay regarding the selectivity of PDE inhibitors, our data clearly show that the rank order of PDE activity in cultured RASMCs is PDE4> PDE1  =  PDE3. The PDE2 activity is absent, and the PDE7 activity uncertain.

In the second part of the work, we analyzed the functional contribution of these PDE isoforms in controlling intracellular cAMP concentration. For this purpose, we took advantage of the FRET technique using an Epac1-based sensor which allows a real-time monitoring of cAMP concentrations in living cells [13], [14]. This approach has been applied extensively in several cell types, including cell lines [14] or freshly isolated cells such as cardiomyocytes [34], but rarely in vascular smooth muscle cells [35], [36]. In mice aorta SMCs maintained one week in culture after dissociation, Iso increased intracellular cAMP with an EC_50_ of about 40 nM [36]. In our study, we also used the physiological β-AR stimulation as cAMP-elevating agent. Cumulative application of Iso produced a significant rise in [cAMP]_i_, with an EC_50_ value of about 18 nM which is in the same range of concentration as that related to its functional relaxant effect in intact rat aorta [37]. Furthermore, both β_1_- and β_2_-AR subtypes contributed to this response, the β_2_ subtype being predominant according to the efficiency of the selective β_1_- and β_2_-AR antagonists to inhibit this response. This is consistent with the fact that both β_1_- and β_2_-AR are localized at the SMC membrane and contribute to the β-adrenergic relaxation in rat aorta [38], [39]. This also indicates that our culture protocol maintains the expression of these receptors [40]. We did not explore the role of the β_3_-AR as this subtype has been shown to be expressed and functional in endothelial cells of rat aorta [37], [41].

A short β-AR stimulation of RASMCs during 15 s induced a fast but transient increase in the cytosolic cAMP concentration with a return to baseline in about 200 s. This is similar to what was observed in other cell types, including rat ventricular cardiomyocytes [34]. The broad spectrum PDE inhibitor, IBMX, markedly prolonged the Iso-triggered cAMP signal, by increasing the t_1/2_off by almost 800%, demonstrating that PDEs play a key role in modulating cytosolic cAMP concentrations in RASMCs. However, even in the presence of IBMX, the cAMP signal was not stable in time and slowly decreased. Several hypotheses might be proposed to explain this observation: (1) the failure of IBMX to totally block PDEs; (2) the role of an IBMX-insensitive PDE isoform in cAMP degradation, such as PDE8; accordingly, we detected PDE8A mRNA in RASMCs. However, we were unable to characterize PDE8 function in our model as no selective PDE8 inhibitor is commercially available; (3) the decrease in cAMP concentration due to its extrusion out of the cell through a multidrug resistance-associated protein, the MRP-4, which inhibition has been shown to slightly increase cAMP concentration in response to forskolin in RASMCs [35]; and (4) the biophysical properties of the Epac1-camps sensor [13], as the decrease of the signal in the presence of IBMX was slowed down when a different Epac-based cAMP sensor was used, the Epac2-camps sensor, characterized by a two times higher affinity than Epac1-camps [13]. Nevertheless, the strong effect of IBMX demonstrates that IBMX-sensitive PDEs play a major role in regulating the dynamic of the β-adrenergic cytosolic cAMP signal.

The PDE subtypes involved in this effect were further characterized using the PDE inhibitors at the concentrations established in the PDE activity assay. We observed that: (1) PDE4 was the main isoform hydrolyzing the cytosolic cAMP produced after β-AR stimulation in RASMCs; (2) PDE1 and PDE3 also contributed to this activity but only when PDE4 was inhibited; (3) PDE2 and PDE7 were absent or inactive. This functional PDE activity pattern is in perfect adequacy with the biochemical PDE activity pattern described above in the same cells, PDE4 being the main active and functional isoform. Furthermore, we observed that PDE4 similarly controlled the cytosolic cAMP pool produced by both β_1_- and β_2_-AR stimulations in RASMCs. This is in agreement with studies showing that PDE4 regulates both β_1_- and β_2_-AR signaling in heterologous expression systems as well as native cardiomyocytes [42], [43]. However, the nature of the PDE4 isoform and the mode of interaction between PDE4 and β-AR subtypes remain to be identified. Interestingly, we showed that PDE4 masked the functional contribution of both PDE1 and PDE3 which was revealed only upon PDE4 inhibition. One explanation might be that under PDE4 inhibition, PDE1 and PDE3 activities are efficiently enhanced to a level allowing them to hydrolyze cAMP in living cells. In fact, PDE3 activity is known to be increased by PKA phosphorylation [44] that might occur following the cAMP increase induced by PDE4 inhibition. However, an opposite regulation pathway is described for PDE1, as its phosphorylation by PKA is known to decrease its activity [3], suggesting that this hypothesis is unlikely, at least for PDE1. A more relevant explanation is that PDE4 confines the cytosolic cAMP generated under β-AR stimulation into a subcellular compartment devoid of PDE1 and PDE3, and that PDE4 inhibition allows cAMP to diffuse in other subcellular compartments enriched in PDE1 and PDE3. The role of PDEs in shaping enzymatic barriers to limit cAMP diffusion has been extensively studied, particularly in cardiomyocytes [6].

To get some insight on the subcellular cAMP compartmentation in our cellular model, we measured cAMP underneath the plasma membrane. To do this, we used a FRET-based sensor which was modified by adding to its N-terminal the “SH4” motif of Lyn kinase. We confirmed that this modification targeted the sensor to the plasma membrane of RASMCs, as previously published in HEK cells [22]. We observed that PDE3 and PDE4 acted in a synergistic manner to hydrolyze the submembrane cAMP produced by β-AR stimulation in RASMCs. This is clearly different from what happened in the cytosolic compartment, suggesting that PDEs delineate distinct subcellular cAMP pools. However, these results should to be interpreted with caution because of some differences between the cytosolic and the plasma membrane-targeted sensors. First, the cytosolic and plasma membrane-targeted sensors operate under 2 different vectors of expression, namely a plasmid and an adenovirus, respectively. This implies appropriate culture conditions for each vector, that might be associated with distinct phenotypes [45]. Second, the two sensors possess distinct Epac-derived cAMP-binding domains, Epac1 and Epac2, which are known to exhibit different affinity for cAMP [13], [22].

In conclusion, our study underlines the importance of cAMP-PDEs for the spatiotemporal control of intracellular cAMP in synthetic RASMCs, and demonstrates the prominent role of PDE4 in the control of both β_1_- and β_2_-AR responses. The synthetic phenotype of SMCs is representative of the phenotype of proliferative SMCs observed in vascular diseases, like atherosclerosis and restenosis [46], and cAMP exerts anti-proliferative and anti-migratory properties in vascular smooth muscle cells. Thus, a therapeutic strategy to counter the pathological vascular proliferative state is to increase cAMP concentrations in these cells. Our data suggest that in this context, a combination of a PDE4 inhibitor with a PDE1 or PDE3 inhibitor would be more potent than a single inhibitor. Furthermore, the effectors regulated by cAMP differ regarding the SMC phenotype (synthetic *versus* contractile). It is thus tempting to speculate that PDE isoforms contribute to the functional specificity of cAMP by delineating distinct subcellular cAMP pools involved in distinct physiological processes. Further work is needed to explore this hypothesis.

## Supporting Information

Figure S1 **Synthetic phenotype of cultured RASMCs.** Immunolabeling of cultured RASMCs with antibodies specific for the α-smooth muscle actin (clone 1A4, Sigma) used as a marker of SMC (***A***), the smooth muscle myosin heavy chain (SMMS-1, Dako) used as a marker of the contractile phenotype of SMC (***B***) or the non-muscle myosin heavy chain (ab684, Abcam) used as a marker of the synthetic phenotype of SMC (***C***). Bar  = 20 µm.
(PPT)

Figure S2 **Effect of PDE1+PDE4 inhibition on β-AR-induced cytosolic cAMP signal in RASMCs.** Cytosolic cAMP measurements were conducted in cultured RASMCs cells using the FRET-based cAMP sensor Epac1-camps in response to a short application of isoproterenol (Iso, 0.1 µM, 15 s) after a pre-treatment in the absence (□) or presence (▪) of 10 µM MIMX + 10 µM Ro. *Top* and *lower panels* represent the mean variation of CFP/YFP ratio and the corresponding kinetic parameters, respectively. Data are mean±SEM of 10–15 independent cells as indicated. ** P<0.01, *** P<0.001 *versus* Iso.
(PPT)

Figure S3 **Effect of the β_1_-AR antagonist on β_2_-AR-induced cytosolic cAMP signals in cultured RASMCs.** Cytosolic cAMP measurements were conducted using the FRET-based cAMP sensor Epac1-camps in response to a maintained application of the β_2_-AR agonist formoterol (0.1 µM, 3 min) after a pre-treatment in the absence or presence of the β_1_-AR antagonist (100 nM CGP-20712A). Data are mean±SEM of 4 independent cells.
(PPT)

Figure S4 **Effect of different concentrations of the β_2_-AR antagonist on β-AR-induced cytosolic cAMP signals in cultured RASMCs.** Cytosolic cAMP measurements were conducted using the FRET-based cAMP sensor Epac1-camps in response to a short application of isoproterenol (Iso, 0.1 µM, 15 s) after a pre-treatment in the absence or presence of increasing concentrations of the β_2_-AR antagonist (1, 5, 10 and 100 nM ICI 118,551, *ICI*). The peak amplitude of the FRET-signal obtained in the different conditions is expressed in % of the isoproterenol-induced response (*Control*). Data are mean±SEM of 4–9 independent cells. * P<0.05 *versus* Iso.
(PPT)
