# Supplementary material for: β-Adrenergic cAMP Signals Are Predominantly Regulated by Phosphodiesterase Type 4 in Cultured Adult Rat Aortic Smooth Muscle Cells
Source: PLoS One. 2012 Oct 18;7(10):e47826. doi: 10.1371/journal.pone.0047826 (PMC3475707; doi:10.1371/journal.pone.0047826)
Supplement: Figure S1 — Synthetic phenotype of cultured RASMCs. Immunolabeling of cultured RASMCs with antibodies specific for the α-smooth muscle actin (clone 1A4, Sigma) used as a marker of SMC (A), the smooth muscle myosin heavy chain (SMMS-1, Dako) used as a marker of the contractile phenotype of SMC (B) or the non-muscle myosin heavy chain (ab684, Abcam) used as a marker of the synthetic phenotype of SMC (C). Bar = 20 µm. (PPT) [file pone.0047826.s001.ppt]

## Slide 1
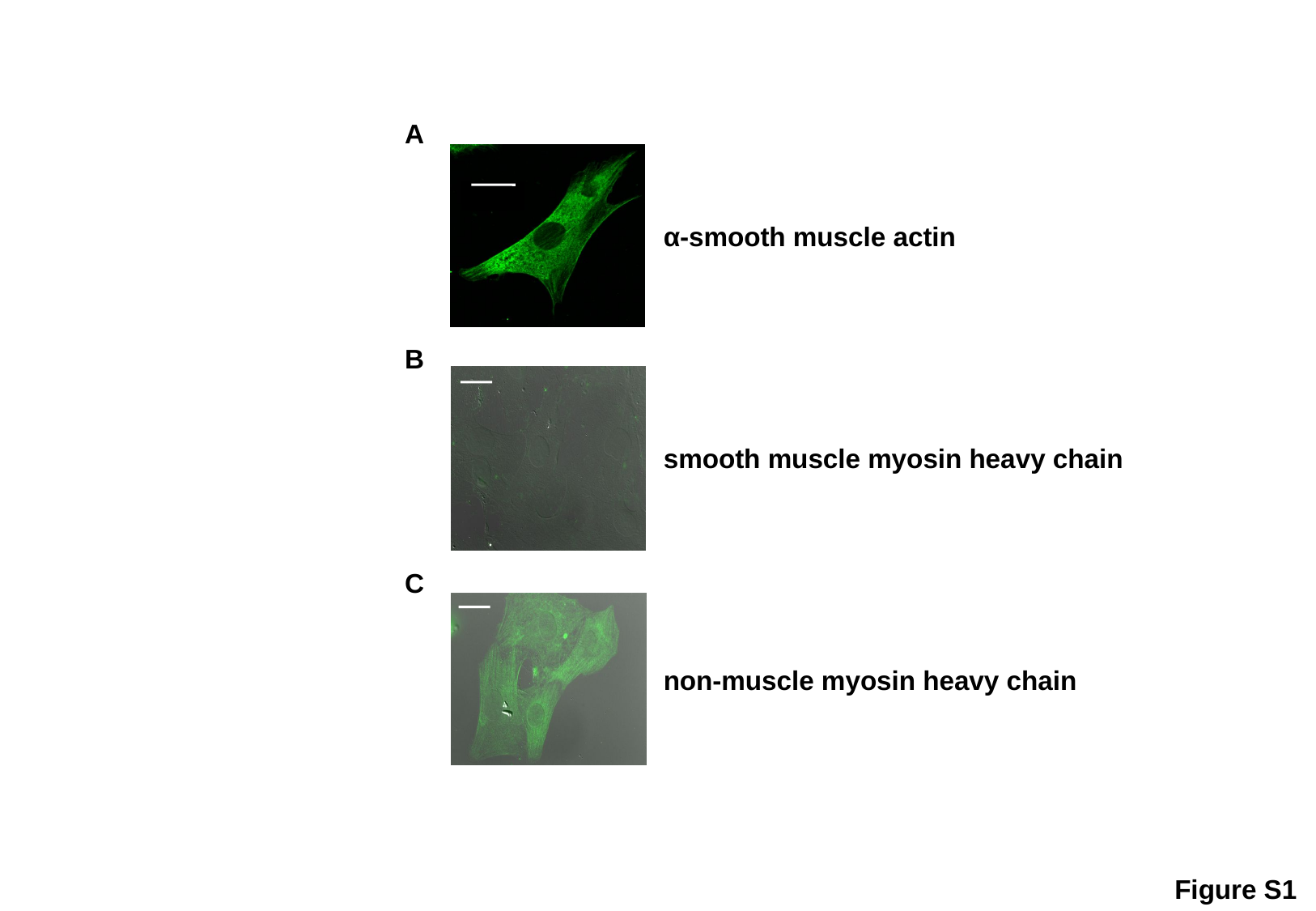

A
20 µm
α-smooth muscle actin
B
smooth muscle myosin heavy chain
C
non-muscle myosin heavy chain
Figure S1
