# Supplementary figures and images for: β-Adrenergic cAMP Signals Are Predominantly Regulated by Phosphodiesterase Type 4 in Cultured Adult Rat Aortic Smooth Muscle Cells
Source: PLoS One. 2012 Oct 18;7(10):e47826. doi: 10.1371/journal.pone.0047826 (PMC3475707; doi:10.1371/journal.pone.0047826)

## Slide 1
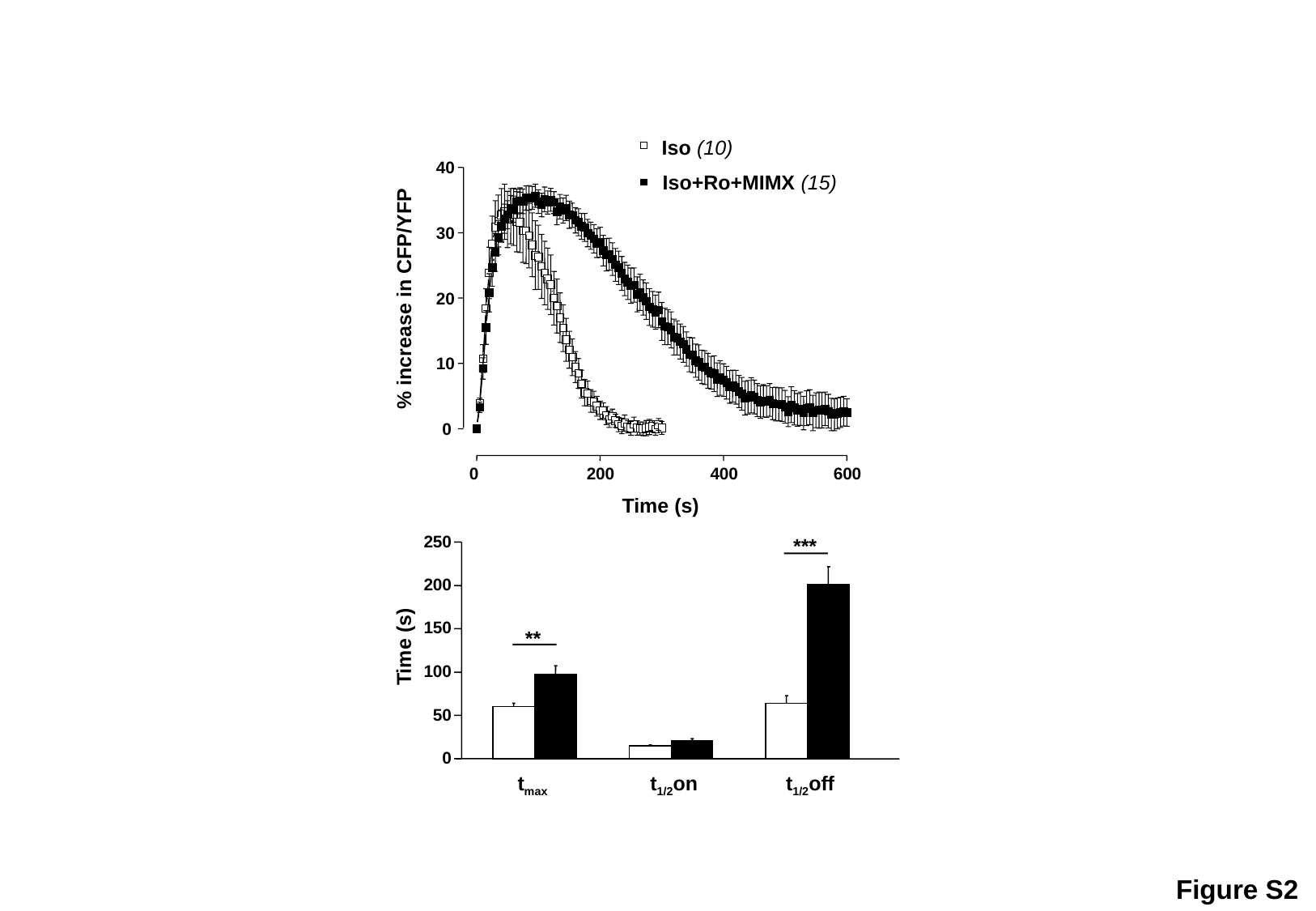

Iso (10)
40
 Iso+Ro+MIMX (15)
30
% increase in CFP/YFP
20
10
0
0
200
400
600
Time (s)
***
250
200
150
**
Time (s)
100
50
0
tmax
t1/2on
t1/2off
Figure S2

Supplement: Figure S2 — Effect of PDE1+PDE4 inhibition on β-AR-induced cytosolic cAMP signal in RASMCs. Cytosolic cAMP measurements were conducted in cultured RASMCs cells using the FRET-based cAMP sensor Epac1-camps in response to a short application of isoproterenol (Iso, 0.1 µM, 15 s) after a pre-treatment in the absence (□) or presence (▪) of 10 µM MIMX + 10 µM Ro. Top and lower panels represent the mean variation of CFP/YFP ratio and the corresponding kinetic parameters, respectively. Data are mean±SEM of 10–15 independent cells as indicated. ** P<0.01, *** P<0.001 versus Iso. (PPT) [file pone.0047826.s002.ppt]

## Slide 1
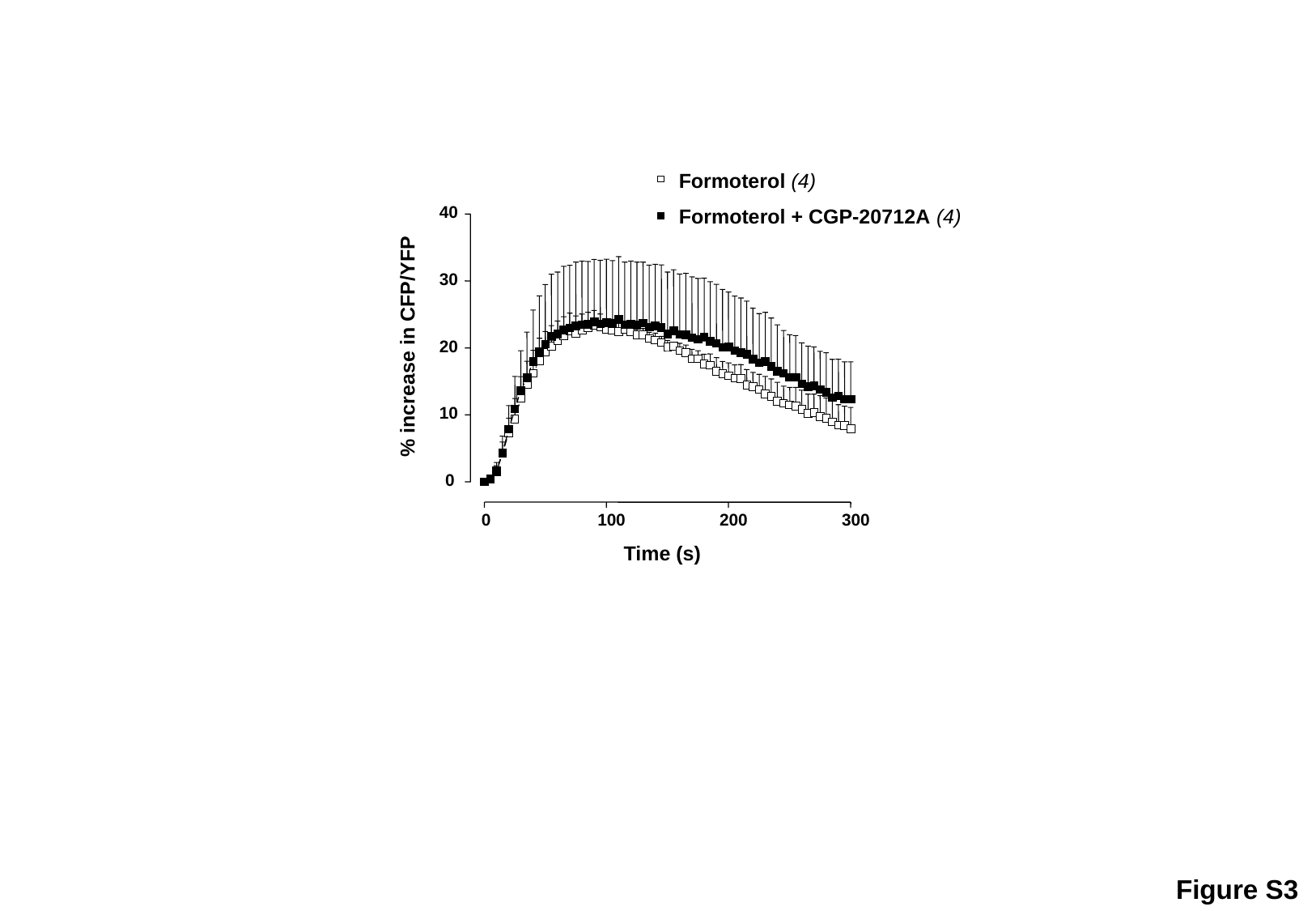

Formoterol (4)
40
 Formoterol + CGP-20712A (4)
30
% increase in CFP/YFP
20
10
0
0
100
200
300
Time (s)
Figure S3

Supplement: Figure S3 — Effect of the β1-AR antagonist on β2-AR-induced cytosolic cAMP signals in cultured RASMCs. Cytosolic cAMP measurements were conducted using the FRET-based cAMP sensor Epac1-camps in response to a maintained application of the β2-AR agonist formoterol (0.1 µM, 3 min) after a pre-treatment in the absence or presence of the β1-AR antagonist (100 nM CGP-20712A). Data are mean±SEM of 4 independent cells. (PPT) [file pone.0047826.s003.ppt]

## Slide 1
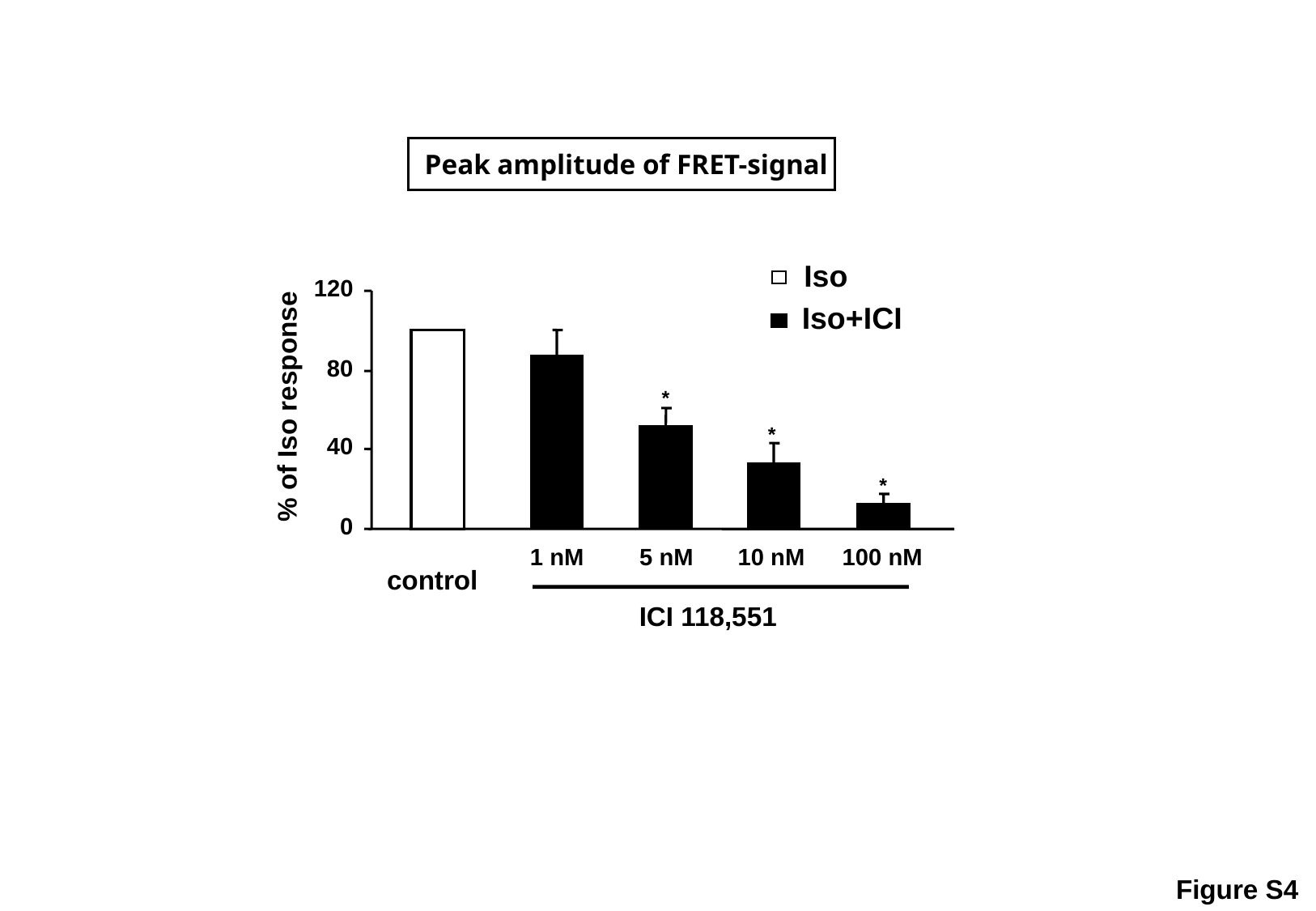

Peak amplitude of FRET-signal
Iso
120
Iso+ICI
80
*
% of Iso response
*
40
*
0
1 nM
5 nM
10 nM
100 nM
control
ICI 118,551
Figure S4

Supplement: Figure S4 — Effect of different concentrations of the β2-AR antagonist on β-AR-induced cytosolic cAMP signals in cultured RASMCs. Cytosolic cAMP measurements were conducted using the FRET-based cAMP sensor Epac1-camps in response to a short application of isoproterenol (Iso, 0.1 µM, 15 s) after a pre-treatment in the absence or presence of increasing concentrations of the β2-AR antagonist (1, 5, 10 and 100 nM ICI 118,551, ICI). The peak amplitude of the FRET-signal obtained in the different conditions is expressed in % of the isoproterenol-induced response (Control). Data are mean±SEM of 4–9 independent cells. * P<0.05 versus Iso. (PPT) [file pone.0047826.s004.ppt]
